# A Sensitive Tg Assay or rhTSH Stimulated Tg: What's the Best in the Long-Term Follow-Up of Patients with Differentiated Thyroid Carcinoma?

**DOI:** 10.1371/journal.pone.0000816

**Published:** 2007-08-29

**Authors:** Adrienne C.M. Persoon, Pieter L. Jager, Wim J. Sluiter, John T.M. Plukker, Bruce H.R. Wolffenbuttel, Thera P. Links

**Affiliations:** 1 Department of Endocrinology, University Medical Centre Groningen, University of Groningen, The Netherlands; 2 Department of Nuclear Medicine, University Medical Centre Groningen, University of Groningen, The Netherlands; 3 Department of Surgery, University Medical Centre Groningen, University of Groningen, The Netherlands; Memorial Sloan-Kettering Cancer Center, United States of America

## Abstract

Sensitivity of thyroglobulin (Tg) measurement in the follow-up of differentiated thyroid carcinoma (DTC) can be optimized by using a sensitive Tg assay and rhTSH stimulation. We evaluated the diagnostic yield of a sensitive Tg assay and rhTSH stimulated Tg in the detection of recurrences in the follow-up of DTC. Additionally the value of imaging techniques for the localization of recurrences was evaluated. We included 121 disease free patients in long-term follow-up for DTC (median 10 years, range 1–34). Tg during thyroid hormone suppression therapy (Tg-on) and rhTSH stimulated Tg were measured with a sensitive Tg assay. Patients with rhTSH stimulated Tg ≥1.0 ng/ml underwent imaging with neck ultrasound, FDG-PET and post therapy ^131^I WBS. Sensitive Tg measurement resulted in 3 patients with Tg-on ≥1.0 ng/ml, recurrence could be localized in 2 of them. RhTSH stimulation resulted in Tg ≥1.0 ng/ml in another 17 of 118 patients. Recurrence could be localized in only 1 additional patient (1 out of 118 patients). Recurrence was localized by neck ultrasound in 1 of 3, by FDG-PET in 2 of 3 and by post therapy ^131^I WBS in 2 of 3 patients. In the detection of recurrences in DTC, rhTSH stimulation had very limited additional value in comparison to Tg-on measurement with a sensitive Tg assay. We consider this too low to justify rhTSH stimulation in all patients during long-term follow up. Neck ultrasound, FDG-PET and post therapy ^131^I WBS showed complementary value in localization of disease, but were only positive in a small fracture of all procedures.

## Introduction

Although differentiated thyroid cancer (DTC) is characterized by an excellent prognosis after thyroidectomy and radioiodine ablation, recurrences occur often even many years after this initial therapy [1]. Therefore, life long follow-up of DTC is recommended. The clinical and economical relevance of optimal follow-up with accurate diagnostic testing is crucial considering the large population of patients in life long follow-up for DTC world-wide.

Traditionally, the cornerstone of follow-up is serum thyroglobulin (Tg) measurement. Optimal sensitivity is reached when Tg is measured during thyrotropin (TSH) stimulation because TSH promotes Tg synthesis [2]. TSH stimulation can be simply achieved by induction of hypothyroidism following thyroid hormone withdrawal. However, externally administered recombinant human TSH (rhTSH) has recently been proven to be an effective alternative to thyroid hormone withdrawal. It stimulates the release of Tg by thyroid remnants and metastatic lesions of DTC without inducing the poorly tolerated side effects of hypothyroidism [3], [4], [5]. Besides the availability of rhTSH, also the recent development of more sensitive Tg assays may considerably increase the sensitivity of Tg measurement to detect recurrent disease. New immunometric assays (IMA) have a functional sensitivity as low as 0.1 ng/ml [6], possibly allowing the detection of smaller amounts of thyroid tissue, even when TSH is suppressed [7].

The current European guidelines for the follow-up of DTC [8] recommend the use of a sensitive Tg assay that is standardized on the European reference standard (CRM 457) [9], [10] with a functional sensitivity <1.0 ng/ml. Additionally, the use of rhTSH for Tg measurement is recommended for the first evaluation 6–12 months after initial therapy to confirm the adequacy of thyroidectomy and radioiodine ablation [8], [11], [12]. Although several studies [4], [5] support the diagnostic accuracy of rhTSH stimulated Tg measurement at the time of this first evaluation, the optimal management of DTC patients concerning Tg measurement in the long-term follow-up phase is less clear. Some authors consider Tg measurement during thyroid hormone suppression therapy (Tg-on) sufficient for the detection of recurrences in low risk patients as long as Tg remains undetectable [7], [8], [13]. Others favour periodic rhTSH stimulated Tg measurement, arguing that tumour may exist in patients with an undetectable Tg-on [11], [12]. Prospective data about these issues are lacking. Accordingly, the recently published American Thyroid Association (ATA) management guidelines for patients with differentiated thyroid cancer state that the timing or necessity of subsequent rhTSH stimulated Tg testing is uncertain for those found to be free of disease [14].

Recently, Smallridge et al. [6] questioned if a Tg assay with improved sensitivity could eliminate the need for rhTSH stimulation when Tg during thyroid hormone suppression therapy is <1.0 ng/ml. This retrospective study showed that when using a sufficiently sensitive Tg assay, these patients rarely have a rhTSH stimulated Tg>2 ng/ml and only one of 80 patients had detectable disease. This recurrence was demonstrated by ultrasound despite numerous imaging studies. Additionally, the lack of localizable disease in a significant part of patients with detectable stimulated Tg, brings up for discussion, whether this stimulation is useful. The lack of evidence that the early detection of especially local disease will improve prognosis actualizes this question even more [15], [16].

For the detection of recurrent disease several imaging methods are used. Neck ultrasound has established a fixed place in the follow-up of DTC, particularly for the detection of cervical lymph node metastasis [17]. The timing and the method of choice for additional imaging is less clear. Applied methods are post therapy ^131^I whole body scan (WBS), CT scan of the neck and chest, FDG-PET scan, and MRI.

In this study we evaluated the introduction of a highly sensitive Tg assay [18] and the additional value of rhTSH stimulation, for the detection of recurrences in disease free patients in long-term follow-up for DTC. Additionally, we evaluated the value of different imaging techniques including neck ultrasound, FDG-PET scan and post therapy ^131^I WBS in the localization of recurrent disease in Tg positive patients.

## Methods

### Study Population

All patients who were treated and followed for DTC in the University Medical Centre Groningen (UMCG) between 1978 and 2003 and were disease free were eligible for participation. Disease free was defined as no clinical evidence of recurrent or persistent DTC and undetectable Tg-on for at least 1 year. This Tg level had been routinely measured by an immunoradiometric assay (CIS Bio International, Gif-sur-Yvette, France) with a detection limit of 1.5 ng/ml for many years. Exclusion-criteria were age under 18 years or above 75 years, pregnancy and severe psychiatric illness. Additionally, patients testing positive for Tg antibodies were excluded.

All patients had undergone initial treatment consisting of total thyroidectomy, followed by radioiodine ablation therapy. Additional treatment, such as lymph node dissection and additional ^131^I therapy was performed when indicated. Routine follow-up included visits at the outpatient clinic every four months in the first two years, and thereafter annually. At each visit physical examination of the neck, Tg-on and TSH measurement were performed. Patients used a suppressive dose of levothyroxin. All patients gave written informed consent to participate in the study, which was approved by the medical ethical review committee of the University Medical Centre Groningen.

### Study design

At baseline, Tg-on was measured with a new sensitive Tg assay (see “Serum measurements” section) in all patients. Subsequently, all patients underwent rhTSH stimulated Tg measurement. Patients with rhTSH stimulated Tg ≥1.0 ng/ml underwent additional imaging including FDG-PET scanning, neck ultrasound and post therapy ^131^I BS to detect recurrent disease. After 3 months Tg-on and rhTSH stimulated Tg measurement were repeated.

#### RhTSH stimulated Tg measurement

RhTSH (Thyrogen, Genzyme Corporation, Cambridge, MA) was administered intramuscularly (i.m) at a dose of 0.9 mg once a day for two consecutive days, while maintaining thyroid hormone suppression therapy. Seventy-two hours after the second rhTSH injection, Tg and TSH were measured.

#### Imaging studies and radioiodine treatment

For detection of recurrent disease, patients underwent 18-F Fluorodeoxyglucose (FDG) Positron Emission Tomography (PET), ultrasound examination of the neck (with fine needle aspiration when indicated) and treatment with 150 mCurie ^131^I followed by a post therapy ^131^I whole body scan (WBS).

FDG-PET scan was performed during thyroid hormone withdrawal, using an ECAT HR+ camera (Siemens/CTI, Knoxville, TN). Patients fasted overnight before the investigation. Ninety minutes after intravenous injection of 5 MBq/kg of FDG, a 2-D whole-body image was acquired from top of the skull to the knees. Images were reconstructed using iterative methods with attenuation correction. Emission time was 5 minutes and transmission time was 3 minutes.

Ultrasound of the neck was performed by an experienced and dedicated radiologist using a Siemens machine with a linear 13 MHz transducer, with fine needle aspiration of suspicious lymph nodes or masses. Lymph nodes with short axis ≥10 mm and/or round oval shape, unsharp borders, inhomogeneous pattern (in particular calcifications or cystic changes), absence of echogenic hilus or hypoechoic pattern [17], [19], [20] were biopsied with percutaneous fine needle aspiration. In case of a hot spot in the neck on the FDG-PET scan, this information was passed to the radiologist who tried to puncture this specific node.

Post therapy ^131^I WBS was performed with a two-headed gammacamera (Multispect 2, Siemens) with a high-energy collimator. We acquired three to four 10-minute adjacent spot views covering the whole body in anterior and posterior views. When necessary, additional views were obtained. The post therapy ^131^I WBS was performed 10 days after the administration of 150 mCurie ^131^I. All these imaging procedures were performed within two weeks.

Additional imaging, including magnetic resonance imaging (MRI) and computed tomography (CT) imaging was performed to confirm positive findings on either post therapy 131I WBS or FDG-PET scan. Levothyroxin was stopped six weeks before radioiodine treatment and replaced by triiodothyronine until two weeks before radioiodine treatment. Levothyroxin was restarted after the treatment. A low iodine diet was followed during one week before treatment. Tg and TSH were measured six weeks after thyroid hormone withdrawal (Tg-off), on the day of the 131I treatment.

To evaluate the effect of radioiodine treatment, we repeated Tg-on and rhTSH stimulated Tg measurement four months after treatment with 150 mCurie ^131^I.

#### Evaluation of imaging and final disease status

All imaging, radiological and nuclear images were evaluated and compared with previous scans by an independent expert panel consisting of endocrinologists, nuclear medicine physicians and a head-neck surgeon, aided by a radiologist when required. Disease status of each patient was assessed and categorized as “no recurrence localized” or “recurrence localized”. “Recurrence localized” implied one or more abnormal imaging studies strongly suggestive of recurrent or metastatic thyroid carcinoma. “No recurrence localized” implied negative radiological and nuclear imaging. Subsequently, the expert panel determined the therapeutic consequences of the findings.

### Serum measurements

Before inclusion in this study, during routine follow-up, Tg was measured by an immunoradiometric assay with functional sensitivity of 1.5 ng/ml (Cis Bio International, Gif-sur-Yvette, France). During the study, Tg was measured by a more sensitive assay (Nichols Advantage® Tg assay, Nichols Institute Diagnostics, San Clement, CA, USA). This is a fully automated chemiluminescence sandwich immunoassay with functional sensitivity of 0.6 ng/ml and calibrated against the CRM-457 reference preparation [18].

TSH was measured by a time-resolved fluoroimmunoassay using the DELFIA system (PerkinElmer Life Sciences, Turku, Finland) with a detection limit of 0.003 mU/l. Tgab were measured by a chemiluminescence immunoassay (Nichols Advantage, Nichols Institute Diagnostics, San Clement, CA, USA) with a cut-off value for TgAb positivity of 2 IU/ml [21]. All serum measurements were performed in the same institution (UMCG).

### Statistical analysis

Data are expressed as median and range. The differences in Tg and TSH before and after radioiodine treatment were analysed using the Wilcoxon test for paired data. For statistical reasons, Tg values <0.6 were considered to be equal to 0.6 ng/ml and TSH values <0.003 were considered to be equal to 0.003 mU/L in this test. P-values of less than 0.05 were considered to indicate statistical significance. Statistical analysis was performed using SPSS version 10.0 software (SPSS, Inc., Chicago, ΙL.).

## Results

### Study patients

Hundred twenty-one patients with DTC (female 76%, median age 54 years, median follow-up 10 years (range 1–34) after initial surgery) were studied (Table 1). Patients were divided in three groups on the basis of Tg-on result.

**Table 1:** Disease characteristics.

Characteristic	All patients N = 121	Patients with undetectable Tg-on N = 115	Patients with Tg-on 0.6-1.0 ng/ml N = 3	Patients with Tg-on≥1.0 ng/ml N = 3
Sex–*no. (%)*				
Female	92 *(76)*	89 *(77)*	2 *(67)*	1 *(67)*
Male	29 *(24)*	26 *(13)*	1 *(33)*	2 *(33)*
Age^a^ (yrs)	54 *(43–61)*	54 *(43–61)*	49 *(33–51)*	56 *(31–64)*
Histology–*no. (%)*				
Papillary	86 *(71)*	81 *(70)*	3 *(100)*	2 *(67)*
Follicular	29 *(24)*	29 *(25)*	0	0
Hürthle cell	6 *(5)*	5 *(4)*	0	1 *(33)*
Follow-up^a^ (yrs)	10 *(6–16)*	10 *(6–16)*	8 *(5–27)*	16 *(5–23)*
TNM-classification^b^–*no. (%)*				
T1-T3	100 *(82)*	96 *(84)*	3 *(100)*	1 *(33)*
T4	3 *(3)*	2 *(2)*	0	1 *(33)*
Tx	18 *(15)*	17 *(15)*	0	1 *(33)*
N0	85 *(70)*	84 *(73)*	1 *(33)*	0
N1	32 *(27)*	27 *(24)*	2 *(67)*	3 *(100)*
Nx	4 *(3)*	4 *(4)*	0	0
M0	120 *(99)*	114 *(99)*	3 *(100)*	3 *(100)*
M1	1 *(1)*	1 *(1)*	0	0
TNM-stage^b^–*no. (%)*				
<45 years				
stage I	72 *(60)*	68 *(59)*	3 *(100)*	1 *(33)*
stage II	0	0	0	0
>45 years				
stage I	5 *(4)*	5 *(4)*	0	0
stage II	26 *(21)*	26 *(23)*	0	0
stage III	11 *(9)*	9 *(8)*	0	2 *(67)*
stage IV	1 *(1)*	1 *(1)*	0	0
unknown	6 *(5)*	6 *(5)*	0	0
Risk-group^c^–*no. (%)*				
Low-risk	103 *(85)*	99 *(86)*	3 *(100)*	1 *(33)*
High-risk	12 *(10)*	10 *(9)*	0	2 *(67)*
Unknown	6 *(5)*	6 *(5)*	0	0

a Data are medians with interquartile range (25^th^ and 75^th^ centile) in the groups “All patients, n = 121” and “Patients with undetectable Tg-Tg-on”, N = 115”.

Data are medians and range in the groups “Patients with Tg-on 0.6–1.0 ng/ml, N = 3”and “Patients with Tg-on >1.0, N = 3”.

b TNM-classification and staging according to Hermanek & Sobin, 1992 [34].

c Low-risk patients are stage I disease if younger than 45 years or Stage I or II if older than 45 years.

#### Group 1: Patients with Tg-on ≥1.0 ng/ml

In three patients Tg-on was ≥1.0 ng/ml (table 2). As expected, in these three patients Tg after rhTSH was also ≥1.0 ng/ml. Imaging resulted in the localization of a recurrence in two of them. In patient A (Tg-on 1.1 ng/ml) no recurrence could be identified on FDG-PET scan, neck ultrasound, post therapy ^131^I WBS or MRI scan of the neck region (Table 3). In patient B (Tg-on 1.3 ng/ml), recurrence was located in the lower jugular region and identified on neck ultrasound, MRI and CT scan. There was no ^131^I uptake. Selective neck dissection was performed and three malignant lymph nodes were removed. Patient C (Tg-on 2.8 ng/ml) had an extensive paravertebral/mediastinal recurrence, identified on FDG-PET scan, post therapy ^131^I WBS, and a subsequent MRI, CT scan and octreotide scintigram. This tumour was inoperable and not amenable to radioiodine or octreotide therapy. External radiotherapy with palliative intention was started. Patient died from respiratory insufficiency due to the consequences of metastatic thyroid carcinoma, eleven months after the identification of recurrent disease.

**Table 2:** Serum Tg and TSH levels.

	Patients with Tg-on ≥1.0 ng/ml a (N = 3)	Patients with Tg-on 0.6-1.0 ng/ml a (N = 3)	Patients with Tg-on <0.6 ng/ml g (N = 115)
Tg-on (ng/ml) b	1.3 (1.1–2.8)	0.81 (0.76–0.99)	<0.6
TSH (mU/l)	0.04 (0.004–0.075)	0.014 (0.003–2.4)	0.048 (0.017–0.31)
Tg after rhTSH (ng/ml)	5.3 (4.5–6.7)	1.3 (<0.6–8.6)	<0.6 (<0.6–<0.6)
TSH (mU/l)	13 (9.2–19)	7.4 (14.0–15.0)	14.0 (9.8–20.0)
Tg-off (ng/ml) c	8.1 (3.5–10.0)	2.6/20.0 e	3.75 (1.65–6.78) f
TSH (mU/l)	16.0 (21.0–46.0)	48.0/50.0	46.5 (35.5–53.5)
Tg-on (ng/ml), 4 months after radioiodine treatment	1.0 (0.84–1.9)	<0.6/2.6 e	<0.6 (<0.6–<0.6) f
TSH (mU/l)	0.018 (0.014–0.022)	0.019/0.11	0.03 (0.008–0.46)
Tg after rhTSH (ng/ml), 4 months after radioiodine treatment	5.3^d^	0.96/10.0 e	1.35 (0.99–1.95) f
TSH (mU/l)	26	17/26	14.5 (9.9–21.5)

a Data are medians with range.

b Tg-on: Tg during thyroid hormone suppression therapy.

c Tg-off: Tg after 6 weeks thyroid hormone withdrawal.

d N = 1: RhTSH stimulated Tg measurement, 4 months after radioiodine treatment was performed in one of three patients. One patient refused, the second patient was not stimulated because of extensive disease.

e N = 2: RhTSH stimulated Tg was ≥1.0 ng/ml in 2 patients, these patients were referred for imaging.

f N = 14: RhTSH stimulated Tg was ≥1.0 ng/ml in 15 patients, imaging was performed in 14 patients. In one patient imaging was not performed because of pregnancy wish.

g Data are medians with interquartile range (25^th^ and 75^th^ centile)

**Table 3:** Patients with Tg-on ≥1.0 ng/ml.

Pt	Age/Sex^a^	Histology^b^	TNM^c^	Risk group^c^	Follow-up (yrs)	Tg-on^d^,^e^	Tg after rhTSH^e^	Tg-off^e^,^f^	Tg-on 4 months after I^131 ^therapy^e^	Tg after rhTSH 4 months after I^131 ^therapy^e^	Evaluation of imaging	Final disease status^h^	Therapeutic consequences
**A**	56/M	Pap	2 1 0	Low-risk	23	1.1	4.5	8.1	NP^g^	NP^g^	Negative	NRL	None
**B**	31/F	Pap	4 1 0	Low-risk	5	1.3	6.7	10.0	0.84	5.3	**Neck lesion**: identified on neck US, MRI and CT	**Recurrence**	Surgical exploration
**C**	64/M	Hürthle	x 1 0	High-risk	16	2.8	5.3	4.7	1.9	NP^g^	**Mediastinal/paravertebral lesion**: identified on FDG-PET, posttherapy I^131 ^WBS, CT, MRI, and Octreotide.	**Recurrence**	Radiotherapy palliation

a M: male, F:female.

b Pap: papillary, Foll: follicular, Hürthle: Hürthle cell.

c TNM, TNM classification and risk group staging [34].

d Tg-on: Tg during thyroid hormone suppression therapy.

e All Tg results in ng/ml.

f Tg-off: Tg after thyroid hormone withdrawal.

g NP: not performed.

h Recurrence: recurrence localized, NRL: no recurrence localized.

Tg-on measurement (table 2) was repeated 4 months after radioiodine treatment (before neck dissection in patient B and the start of radiotherapy in patient C, patient A refused Tg measurement). Tg-on was lower but remained detectable in both patients (table 3). Only in patient B rhTSH stimulated Tg measurement was repeated after radioiodine therapy (patient A refused and patient C was not stimulated because of extensive disease). RhTSH stimulated Tg was 6.7 ng/ml before radioiodine therapy and 5.3 ng/ml after radioiodine therapy.

#### Group 2: Patients with Tg-on 0.6–1.0 ng/ml

Three patients (table 2) had detectable Tg-on levels between 0.6 and 1.0 ng/ml (0.76, 0.81 and 0.99 ng/ml). After rhTSH stimulation Tg was undetectable in the first patient and rose to 1.3 and 8.6 ng/ml in the two other patients. These two patients were referred for imaging. In none of them recurrence was localized (table 4).

**Table 4:** Patients with Tg-on 0.6–1.0 ng/ml and rhTSH stimulated Tg ≥1.0 ng/ml.

Pt	Age/Sex^a^	Histology^b^	TNM^c^	Risk group^c^	Follow-up (yrs)	Tg-on^d^,^e^	Tg after rhTSH^e^	Tg-off^e^,^f^	Tg-on 4 months after I^131 ^therapy^e^	Tg after rhTSH 4 months after I^131 ^therapy^e^	Evaluation of imaging	Final disease status^h^	Therapeutic consequences
**D**	51/F	Pap	1 1 0	Low-risk	27	0.81	1.3	2.6	<0.6	0.96	Negative	NRL	None
**E**	33/M	Pap	2 1 0	Low-risk	8	0.99	8.6	20	2.6	10.0	Negative	NRL	None

a M: male, F:female.

b Pap: papillary, Foll: follicular, Hürthle: Hürthle cell.

c TNM, TNM classification and risk group staging [34].

d Tg-on: Tg during thyroid hormone suppression therapy.

e All Tg results in ng/ml.

f Tg-off: Tg after thyroid hormone withdrawal.

g NP: not performed.

h Recurrence: recurrence localized, NRL: no recurrence localized.

Four months after radioiodine treatment Tg-on and rhTSH stimulated Tg were measured again (table 4). Tg-on after radiodine treatment remained detectable in one of both patients (2.6 ng/ml). At the second rhTSH stimulated Tg measurement, Tg remained detectable in both patients (0.96 and 10.0 ng/ml).

#### Group 3: Patients with undetectable Tg-on (<0.6 ng/ml)

Tg-on was undetectable in 115 patients. After rhTSH stimulation, Tg became detectable in 19 patients (table 2). In 15 patients (13%) rhTSH stimulated Tg was ≥1.0 ng/ml (median 1.6 ng/ml, range 1.0–5.4). Imaging was performed in 14 out of these 15 patients with undetectable Tg-on and rhTSH stimulated Tg ≥1.0 ng/ml. One patient refused imaging because of pregnancy wish. After evaluation of all images, recurrence was localized in one of 14 patients (table 5). Recurrence was localized in patient F and was located in the right supraclavicular fossa and identified on FDG-PET scan, post therapy ^131^I WBS and MRI scan. The suspicious lesion was resected and histopathological analysis showed a lymph node metastasis of papillary thyroid carcinoma. In the remaining 13 patient no recurrence could be localized.

**Table 5:** Patients with undtectable Tg-on and rhTSH stimulated Tg ≥1.0 ng/ml.

Pt	Age/Sex^a^	Histology^b^	TNM^c^	Risk group^c^	Follow- up (yrs)	Tg-on^d^,^e^	Tg after rhTSH^e^	Tg-off^e^,^f^	Tg-on 4 months after I^131 ^therapy^e^	Tg after rhTSH 4 months after I^131 ^therapy^e^	Evaluation of imaging	Final disease status^h^	Therapeutic consequences
**F**	58/M	Pap	2 0 0	Low-risk	12	<0.6	3.9	4.8	<0.6	1.9	Supraclavicular lesion: identified on FDG-PET, posttherapy I^131 ^WBS, MRI	Recurrence	Surgical exploration
**G**	55/F	Pap	2 1 0	High-risk	3	<0.6	2.8	6.7	<0.6	1.6	Negative	NRL	None
**H**	50/M	Foll	2 0 0	Low-risk	10	<0.6	3.1	11.0	0.6	2.1	Negative	NRL	None
**I**	39/M	Pap	2 1 0	Low-risk	4	<0.6	3.0	8.4	<0.6	2.5	Negative	NRL	None
**J**	55/F	Pap	2 0 0	Low-risk	10	<0.6	1.2	2.3	<0.6	1.3	Negative	NRL	None
**K**	43/F	Pap	2 0 0	Low-risk	17	<0.6	1.6	6.0	<0.6	0.79	Negative	NRL	None
**L**	36/F	Pap	2 1 0	Low-risk	16	<0.6	2.2	NP^g^	<0.6	NP^g^	No imaging because of pregnancy wish	-	-
**M**	53/F	Pap	1 0 0	Low-risk	3	<0.6	1.0	1.8	<0.6	<0.6	Negative	NRL	None
**N**	60/M	Pap	4 1 0	High-risk	5	<0.6	1.0	1.7	<0.6	1.0	Negative	NRL	None
**O**	68/M	Pap	1 1 0	High-risk	16	<0.6	1.4	1.0	<0.6	1.3	Negative	NRL	None
**P**	55/M	Pap	2 1 0	Low-risk	18	<0.6	1.2	1.5	<0.6	1.2	Negative	NRL	None
**Q**	48/F	Pap	2 0 0	Low-risk	15	<0.6	5.4	2.7	<0.6	4.3	Negative	NRL	None
**R**	42/F	Pap	2 0 0	Low-risk	5	<0.6	1.4	5.1	<0.6	1.4	Negative	NRL	None
**S**	46/F	Pap	4 0 0	Low-risk	18	<0.6	3.9	7.0	<0.6	1.9	Negative	NRL	None
**T**	60/M	Fol	1 1 0	Low-risk	21	<0.6	1.1	0.85	<0.6	0.96	Negative	NRL	None

a M: male, F:female.

b Pap: papillary, Foll: follicular, Hürthle: Hürthle cell.

c TNM, TNM classification and risk group staging [34].

d Tg-on: Tg during thyroid hormone suppression therapy.

e All Tg results in ng/ml.

f Tg-off: Tg after thyroid hormone withdrawal.

g NP: not performed.

h Recurrence: recurrence localized, NRL: no recurrence localized.

Four months after radioiodine treatment (and before surgery in patient F), Tg-on and rhTSH stimulated Tg were measured again (table 2). Tg-on had become detectable in 1 of 14 patients, Tg and TSH levels did not significantly differ (p = 0.4 and 0.7) compared to the situation before radioiodine treatment. At the second rhTSH stimulated Tg measurement, Tg remained detectable in 13 of 14 patients but was significantly lower (p = 0.005) than before radioiodine treatment while no difference in TSH was found (p = 0.084).

#### Diagnostic yield of imaging tests

Imaging studies were performed in 19 of 20 patients with rhTSH stimulated Tg ≥1.0 ng/ml (one patient refused because of pregnancy wish). All these patients underwent neck ultrasound, FDG-PET scan and post therapy^131^I WBS. Additional imaging including MRI, CT and Octreotide scanning was performed when better anatomical localization was needed or when results were contradicting. Only in three patients recurrence could be localized.

In patient B (table 3) recurrence was localized in the neck and was initially detected by neck ultrasound. FDG-PET scan and post therapy^131^I WBS were negative. Exact anatomical localization was achieved by CT and MRI. Patient C (table 3) had extensive metastatic disease, which was visualized by nuclear medicine methods (FDG-PET, radioiodine imaging, octeotride scintigraphy) as well as CT and MRI. Patient F (table 5) with a supraclavicular lesion had a negative ultrasound and positive FDG-PET, post therapy^131^I WBS and MRI.

## Discussion

This prospective study showed that the introduction of a new sensitive Tg assay for clinical disease free patients resulted in the localisation of 2 recurrences (1.8%) in a cohort of 121 patients considered to be in remission. The additional yield of rhTSH stimulation in these patients was 1 additional localized recurrence (1/118 patients, 0.8%). We consider this too low to justify rhTSH stimulation in all patients. These results confirm the retrospective data recently presented by Smallridge et al. [6].

Although the presence of the established risk factors result in a reduced life expectancy [22], also initial low risk patients can die from thyroid cancer [23], illustrating that distinction between high and low risk is limited [24]. Therefore in this study, we included patients irrespective of prognostic factors influencing risk of recurrence. Additionally, most recurrences occur within the first decade after initial therapy, but up to one third of recurrences occur in the subsequent years. Recurrences are described even more than 40 years after initial diagnosis [1]. Therefore, lifelong follow-up is advised [8]. We included patients regardless of follow-up duration. By doing so, this study population consisted of a mixture of low-risk and high-risk patients and variable follow-up duration, reflecting the variety of patients in follow-up for DTC in daily clinical practice [25] and results of this study are applicable to all patients with DTC in follow-up for DTC.

Tg measurement is the cornerstone in the follow-up of DTC but accurate measurement of Tg is technically challenging. An important requirement of Tg assays is a functional sensitivity low enough to detect small amounts of thyroid tissue when TSH is suppressed [7]. The importance and surplus value of a sensitive Tg assay has been shown in this study. Two patients with recurrent disease could be identified solely on the basis of Tg-on levels ≥1.0 ng/ml by using a sensitive Tg assay. In 2 of 3 patients, with Tg-on ≥1.0 ng/ml recurrent disease could be localized so diagnostic yield of imaging was high when using 1.0 ng/ml as cut-off. Tg-on in these patients had been undetectable by the conventional assay, so formerly rhTSH stimulated Tg measurement would have been needed to identify these patients. These patients are illustrative that optimizing sensitivity of Tg assays, may obviate the need for rhTSH stimulated Tg measurement in the future follow-up of DTC [26].

In one patient with undetectable Tg-on, recurrent disease was found. Only in this patient (1 of 121 patients) rhTSH stimulated Tg measurement had additional diagnostic yield in the detection of recurrent disease. Clearly, this very limited result of rhTSH testing does not validate the introduction of routine rhTSH stimulated Tg measurement in the follow-up of DTC. Moreover, regular serial Tg-on measurement with a sensitive Tg assay anyhow would have identified this solely patient, since the change in Tg over time is more informative than a single Tg determination [27]. Tg-on will increase during serial Tg-on measurement [28], [29] when recurrent disease is actually present.

Zanotti-Fregonara et al. [30], also evaluated the utility of rhTSH stimulation. Their results endorse the limited value of periodic rhTSH stimulation in patients with stage I thyroid cancer. In contrast, they recommend rhTSH stimulation in higher-risk patients because of positive Tg levels after rhTSH in 4 of 35 high-risk patients (11%). However, two of these patients already had detectable Tg-on levels. In only one patient, recurrence was localized 10 months after the last I-131 therapy, and according to our definition this patient could not be considered as in remission. Results of our study do not support the use of rTSH stimulation in the long term follow up of high risk patients. Recurrent disease was localized in one low risk patient with undetectable Tg-on after rhTSH stimulation l (patient F) and in one low-risk and one high risk patient using the ultra sensitive Tg assay (patient B and C respectively)

Uncertainty exists about the clinical value of low but detectable Tg-on values in sensitive Tg assays. In the present study a small number of patients had Tg-on levels between 0.6 and 1.0 ng/ml. The rise in Tg after rhTSH to ≥1.0 ng/ml in 2 of these 3 patients indicated residual thyroid tissue and not an assay artefact [31]. Nevertheless, recurrent disease could not be localized. This illustrates that the sensitivity of Tg measurement currently exceeds the sensitivity of the available imaging techniques and therefore careful watching of the slope of Tg-on is recommended. The optimal timing of imaging needs to be ascertained in future studies. Extensive imaging when Tg-on in a sensitive assay rises above a certain cut-off level will be far more efficient, preventing needless patient burden and medical costs.

In the present study, imaging including post therapy ^131^I WBS, neck US and FDG PET was performed in all patients suspected of recurrent disease. Neck ultrasound is considered the most sensitive method to detect local recurrence, although it is not specific and is an operator-dependent procedure [8], [32]. In contrast, radioiodine scanning has high specificity for recurrences of differentiated thyroid cancer but considerably low sensitivity [32]. Particularly in radioiodine negative differentiated thyroid cancer, FDG-PET is useful and has both high sensitivity and specificity [33]. Results of this study showed that these three imaging methods are complementary in the detection of recurrent disease. Nevertheless, additional imaging including CT and MRI scanning was needed to provide better anatomical localization of lesions. This result illustrates that the fusion of anatomy and metabolism by the recently introduced integrated PET/CT systems, is a promising technique [32], [33].

Finding a low or undetectable Tg after rhTSH stimulation could be considered as a reassurance to patients that truly no disease activity is present. In the present study, Tg was <1.0 ng/ml after rhTSH in the vast majority (87%) of patients. Actually, this result only confirmed the already known absence of disease in these patients with undetectable Tg-on. In this group of patients, rhTSH stimulation is an expensive, unnecessary diagnostic test. Moreover, these data underscore the adequacy of undetectable Tg-on confirming the absence of disease and contradict the opinion that “an undetectable serum Tg measured during thyroid hormone suppression is often misleading in a large proportion of patients with residual DTC” [7]. We did not routinely use neck ultrasound during follow-up so we could have missed small lymph node metastases in these patients. For it is known that lymph node metastases can be detected by neck ultrasound in patients with both undetectable Tg-on and undetectable rhTSH stimulated Tg or Tg-off [16], [17]. This is the reason why the combination of Tg measurement and neck ultrasound is now becoming the standard of care, although it is still controversial whether the early detection of generally very small lymph node metastases will improve prognosis [15], [16].

In conclusion, Tg measurement with a sensitive Tg assay has an additional diagnostic yield in the detection of recurrent disease in patients in follow-up for DTC and practically obviates the need for rhTSH stimulated Tg measurement. Long-term follow up of DTC can safely be based on serial Tg-on measurement with a sensitive Tg assay and additional diagnostic tests should be performed only when Tg-on rises above an established cut-off level. This will result in a limited follow up protocol, warranting the detection of recurrences of DTC and reducing patient burden and medical costs.

## References

[1] Mazzaferri EL, Kloos RT (2001). Clinical review 128: Current approaches to primary therapy for papillary and follicular thyroid carcinoma.. J Clin Endocrinol Metab.

[2] Lo Gerfo P, Colacchio TA, Colacchio DA, Feind CR (1980). Effect of TSH stimulation on serum thyroglobulin in metastatic thyroid carcinoma.. J Surg Oncol.

[3] Meier CA, Braverman LE, Ebner SA, Veronikis I, Daniels GH (1994). Diagnostic use of recombinant human thyrotropin in patients with thyroid carcinoma (phase I/II study).. J Clin Endocrinol Metab.

[4] Ladenson PW, Braverman LE, Mazzaferri EL, Brucker-Davis F, Cooper DS (1997). Comparison of administration of recombinant human thyrotropin with withdrawal of thyroid hormone for radioactive iodine scanning in patients with thyroid carcinoma.. N Engl J Med.

[5] Haugen BR, Pacini F, Reiners C, Schlumberger M, Ladenson PW (1999). A comparison of recombinant human thyrotropin and thyroid hormone withdrawal for the detection of thyroid remnant or carcinoma.. J Clin Endocrinol Metab.

[6] Smallridge RC, Meek SE, Morgan MA, Gates GS, Fox TP (2007). Monitoring thyroglobulin in a sensitive immunoassay has comparable sensitivity to recombinant human TSH stimulated thyroglobulin in follow-up of thyroid cancer patients.. J Clin Endocrinol Metab.

[7] Mazzaferri EL, Robbins RJ, Spencer CA, Braverman LE, Pacini F (2003). A consensus report of the role of serum thyroglobulin as a monitoring method for low-risk patients with papillary thyroid carcinoma.. J Clin Endocrinol Metab.

[8] Pacini F, Schlumberger M, Dralle H, Elisei R, Smit JW (2006). European Thyroid Cancer Taskforce. European consensus for the management of patients with differentiated thyroid carcinoma of the follicular epithelium.. Eur J Endocrinol.

[9] Feldt-Rasmussen U, Profilis C, Colinet E, Black E, Bornet H (1996). Human thyroglobulin reference material (CRM 457). 1st Part: Assessment of homogeneity, stability and immunoreactivity.. Ann Biol Clin.

[10] Feldt-Rasmussen U, Profilis C, Colinet E, Black E, Bornet H (1996). Human thyroglobulin reference material (CRM 457). 2nd Part: Physicochemical characterization and certification.. Ann Biol Clin.

[11] Wartofsky L (2002). Using baseline and recombinant human TSH-stimulated Tg measurements to manage thyroid carcinoma without diagnostic (131)I scanning.. J Clin Endocrinol Metab.

[12] Mazzaferri EL, Massoll N (2002). Management of papillary and follicular (differentiated) thyroid carcinoma: new paradigms using recombinant human thyrotropin.. Endocr Relat Cancer.

[13] Schlumberger M, Ricard M, Pacini F (2000). Clinical use of recombinant human TSH in thyroid carcinoma patients.. Eur J Endocrinol.

[14] Cooper DS, Doherty GM, Haugen BR, Kloos RT, Lee SL (2006). Management guidelines for patients with thyroid nodules and differentiated thyroid cancer.. Thyroid.

[15] Torlontano M, Crocetti U, D'Aloiso L, Bonfitto N, Di Giorgio A (2003). Serum thyroglobulin and 131I whole body scan after recombinant human TSH stimulation in the follow-up of low-risk patients with differentiated thyroid cancer.. Eur J Endocrinol.

[16] Torlontano M, Attard M, Crocetti U, Tumino S, Bruno R (2004). Follow-up of low risk patients with papillary thyroid carcinoma: role of neck ultrasonography in detecting lymph node metastases.. J Clin Endocrinol Metab.

[17] Frasoldati A, Pesenti M, Gallo M, Caroggio A, Salvo D (2003). Diagnosis of neck recurrences in patients with differentiated thyroid carcinoma.. Cancer.

[18] Persoon ACM, van den Ouweland JMW, Wilde J, Kema IP, Wolffenbuttel BHR (2006). Clinical utility of an automated immunochemiluminometric thyroglobulin assay in differentiated thyroid carcinoma.. Clin Chem.

[19] Kessler A, Rappaport Y, Blank A, Marmor S, Weiss J (2003). Cystic appearance of cervical lymph nodes is characteristic of metastatic papillary thyroid carcinoma.. J Clin Ultrasound.

[20] Ahuja AT, Ying M, Yuen HY, Metreweli C (2001). Power Doppler sonography of metastatic nodes from papillary carcinoma of the thyroid.. Clin Radiol.

[21] Persoon ACM, Links TP, Wilde J, Sluiter WJ, Wolffenbuttel BHR (2006). Potential contributory value of thyroglobulin (Tg) recovery testing to quantitative Tg antibody measurement in determining serum Tg measurement interference in differentiated thyroid carcinoma.. Clin Chem.

[22] Links TP, van Tol KM, Jager PL, Plukker JT, Piers DA (2005). Life expectancy in differentiated thyroid cancer: a novel approach to survival analysis.. Endocr Relat Cancer.

[23] Eustatia-Rutten CF, Corssmit EP, Biermasz NR, Pereira AM, Romijn JA (2006). Survival and death causes in differentiated thyroid carcinoma.. J Clin Endocrinol Metab.

[24] Links TP, Jager PL, Persoon ACM, Plukker JTM, Sluiter WJ (2006). Age is not a prognostic factor in differentiated thyroid carcinoma.. J Clin Endocrinol Metab. Electronic Letter to the Editor 4 January.

[25] Schlumberger MJ (1998). Papillary and follicular thyroid carcinoma.. N Engl J Med.

[26] Spencer CA, Bergoglio LM, Kazarosyan M, Fatemi S, Lopresti JS (2005). Clinical Impact of Thyroglobulin (Tg) and Tg autoantibody Method Differences on the Management of patients with Differentiated Thyroid Carcinomas.. J Clin Endocrinol Metab.

[27] Spencer CA, LoPresti JS, Fatemi S, Nicoloff JT (1999). Detection of residual and recurrent differentiated thyroid carcinoma by serum thyroglobulin measurement.. Thyroid.

[28] Demers CM, Spencer CA (2002). National Academy of Clinical Biochemistry Laboratory support for the diagnosis and monitoring of thyroid disease.. http://www.nacb.org/lmpg/thyroid_lmpg_pub.stm.

[29] Rosario do PW, Borges MA, Fagundes TA, Franco AC, Purisch S (2005). Is stimulation of thyroglobulin (Tg) useful in low-risk patients with thyroid carcinoma and undetectable Tg on thyroxin and negative neck ultrasound?. Clin Endocrinol.

[30] Zanotti-Fregonara P, Khoury A, Duron F, Keller I, Christin-Maitre S (2007). Which thyroid cancer patients need periodic stimulation tests?. Eur J Nucl Med Mol Imaging.

[31] Morgenthaler NG, Froehlich J, Rendl J, Willnich M, Alonso C (2002). Technical evaluation of a new immunoradiometric and a new immunoluminometric assay for thyroglobulin.. Clin Chem.

[32] Lind P, Kohlfurst S (2006). Respective roles of thyroglobulin, radioiodine imaging, and positron emission tomography in the assessment of thyroid cancer.. Semin Nucl Med.

[33] Nanni C, Rubello D, Fanti S, Farsad M, Ambrosini V (2006). Role of 18F-FDG-PET and PET/CT imaging in thyroid cancer.. Biomed Pharmacother.

[34] Hermanek P, Sobin LH, Hermanek P, Sobin LH (1992). Thyroid gland.. TNM classification of malignant tumours, edn 4.

